# Multiple *Ehrlichia chaffeensis* genes critical for persistent infection in a vertebrate host are identified as nonessential for its growth in the tick vector; *Amblyomma americanum*

**DOI:** 10.3389/fcimb.2023.1220025

**Published:** 2023-06-27

**Authors:** Deborah C. Jaworski, Ying Wang, Arathy Nair, Huitao Liu, Roman R. Ganta

**Affiliations:** ^1^Center of Excellence for Vector-Borne Diseases, Department of Diagnostic Medicine/Pathobiology, College of Veterinary Medicine, Kansas State University, Manhattan, KS, United States; ^2^Department of Veterinary Pathobiology, College of Veterinary Medicine, Bond Life Sciences Center, University of Missouri, Columbia, MO, United States

**Keywords:** *Ehrlichia chaffeensis*, rickettsial genetics, tick-borne pathogens, tick- and host-specific *Ehrlichia* genes, mutational studies

## Abstract

*Ehrlichia chaffeensis* is a tick-transmitted monocytic ehrlichiosis agent primarily causing the disease in people and dogs. We recently described the development and characterization of 55 random mutations in *E. chaffeensis*, which aided in defining the critical nature of many bacterial genes for its growth in a physiologically relevant canine infection model. In the current study, we tested 45 of the mutants for their infectivity ability to the pathogen’s tick vector; *Amblyomma americanum*. Four mutations resulted in the pathogen’s replication deficiency in the tick, similar to the vertebrate host. Mutations causing growth defects in both vertebrate and tick hosts included in genes coding for a predicted alpha/beta hydrolase, a putative dicarboxylate amino acid:cation symporter, a T4SS protein, and predicted membrane-bound proteins. Three mutations caused the bacterial defective growth only in the tick vector, which represented putative membrane proteins. Ten mutations causing no growth defect in the canine host similarly grew well in the tick vector. Mutations in 28 genes/genomic locations causing *E. chaffeensis* growth attenuation in the canine host were recognized as non-essential for its growth in the tick vector. The tick non-essential genes included genes coding for many metabolic pathway- and outer membrane-associated proteins. This study documents novel vector- and host-specific differences in *E. chaffeensis* for its functional gene requirements.

## Introduction

The lone star tick, *Amblyomma americanum*, continues to be one of the most successful ticks during the past several decades and is a major contributor to the rise in documented tick-borne diseases in the USA (Rodino et al., 2020; Higuita et al., 2021). This tick has expanded its range as well as the spectrum of pathogens that it transmits (Higuita et al., 2021). As a three-host tick, all its motile life stages (larva, nymph, and adult) aggressively blood feed on a wide range of hosts, including people and dogs. This tick is the vector for *Ehrlichia chaffeensis* which infects several mammalian hosts, including people, dogs, coyotes, goats, and white-tailed deer (Dawson et al., 1996; Lockhart et al., 1997; Breitschwerdt et al., 1998; Dugan et al., 2000; Kocan et al., 2000; Davidson et al., 2001). This pathogen belongs to the Anaplasmataceae family which includes several obligate intracellular pathogenic bacteria of the genera *Ehrlichia, Anaplasma, Rickettsia* and *Orientia* (Dumler et al., 2001).

For most tick-borne diseases, there is a close interplay between tick vectors, vertebrate hosts and pathogenic organisms transmitted by ticks. Pathogens must navigate both the vertebrate and tick systems to efficiently adapt to the dual hosts for their continued survival despite the immune responses exerted by ticks and vertebrate hosts. In a recent study, we developed and evaluated an *E. chaffeensis* transposon mutant library constituting of 55 mutants to identify genes essential for its persistent growth in its natural vertebrate infection model (Wang et al., 2020). In that study, we reported that *E. chaffeensis* requires many functional genes. They included genes coding for three immunodominant membrane proteins; two 28 kDa outer membrane proteins (P28/OMP) and a 120 kDa surface protein. Mutations in two biotin biosynthesis pathway genes, a fatty acid biosynthesis gene, and a 3-oxoacyl-(acyl-carrier-protein) reductase gene also resulted in the *E. chaffeensis* rapid clearance from the vertebrate host. Mutations near genes coding for DNA repair and protein synthesis pathway proteins also caused rapid clearance of *E. chaffeensis* from a vertebrate host (Wang et al., 2020).

In the current study, we extended the investigations to define which genes/proteins are essential for *E. chaffeensis* during its replication in its tick vector. We investigated 45 of the previously characterized *E. chaffeensis* random mutants to define the impact of the mutations on the bacterial persistence in the tick host. We assessed the infection status of the mutants in *A. americanum* following needle infection and its molting from the nymphal stage to the adult stage. We discovered remarkable differences in the requirements of functional genes of *E. chaffeensis* for growth and persistence in *A. americanum* compared to the canine host. The study aided in identifying genes critical for both vertebrate and tick hosts and those exclusively needed for the bacterial replication in tick or vertebrate hosts.

## Materials and methods

### Ehrlichia chaffeensis *in vitro* cultivation

*Ehrlichia chaffeensis* mutant organisms (Wang et al., 2020) (45 in all) were cultivated in the canine macrophage cell line (DH82) at 37°C with 5% CO_2_ as described earlier (Chen et al., 1995; Cheng and Ganta, 2008). As infection progression in ticks begins with the pathogen transfer during a bloodmeal originating from a vertebrate host (such as from white tailed deer and dogs), we grew mutant organisms in DH82 for use in tick infection experiments.

### Ticks and tick rearing/tick injections

Freshly engorged nymphs of *A. americanum* were obtained from Ecto Services Inc. (Henderson, NC). Within 48 h of dropping from the host, groups of approximately 50 engorged nymphs were washed thoroughly with 10% bleach, then rinsed three times with sterile water and dried on paper towels. The nymphs were then injected with approximately one microliter each of 5 x 10^8^ *E. chaffeensis* mutant organisms using an inoculum syringe with a 27-gauge needle. The injected ticks were transferred to a humidified incubator (95% relative humidity is maintained with a saturated salt solution of potassium sulfate) maintained at room temperature with 14 h of light. These conditions allowed the molting of nymphs to the adult stage in approximately 4-5 weeks.

### DNA isolation and PCR verification of *E. chaffeensis* mutant-injected ticks

Within four weeks of molting, genomic DNAs from 10 ticks each were individually isolated from each group of mutant-infected adult ticks. Tick genomic DNAs were isolated using a DNeasy Blood and Tissue kit as per the manufacturer’s instructions (Qiagen Sciences Inc., Germantown, MD). Purified DNA from each tick was resuspended in 200 µl of DNA elution buffer. Two microliters of DNA derived from each tick were then used for nested PCR analysis targeting to the insertion segment located within each insertion mutant organism and using the Green Taq Master Mix in a 25 µl PCR assay. The first round of amplification was carried out using the forward primer, AmtrF, 5’CTCCTAGAACGATCGCCGCATGCTAGC and the reverse primer, RRG1598, 5’TTATTTGCCGACTACCTTGGTGAT. The second round of amplification was performed using the forward primer, AmtrF2, 5’CGCGCGCACTAACAAGTGCCC and the above listed reverse primer and with 2 µl of the first round PCR product. The annealing temperature for both PCRs was 55°C for 30 sec and extension was carried out at 72°C for 2 mins for a total of 40 cycles. All PCR products were resolved in 0.9% agarose gels with the 1X tris-acetate-EDTA buffer and stained with ethidium bromide. We performed the nested PCRs two independent times to validate the results and using DNA recovered from 10 ticks per mutant group. All assays included PCR positive controls where a known template was added and negative controls containing no template DNA. Further, wild type *E. chaffeensis* infected and non-infected and molted ticks were included to serve as positive and negative controls, respectively.

### Database searching for protein homologies and for finding protein motifs

The identification of open reading frames (ORFs) encoding proteins in *E. chaffeensis* was carried out using the genome entries; GenBank accession numbers CP000236 and NC_007799.1 present at the NCBI database (https://www.ncbi.nlm.nih.gov/). The secondary structure predictions and conserved domain predictions of *E. chaffeensis* proteins were performed using the online sequence analysis tools; SMART protein (http://smart.embl-heidelberg.de/) (Letunic et al., 2020). PRED-TMR was used for transmembrane domain predictions (http://athina.biol.uoa.gr/PRED-TMR/) (Pasquier et al., 1999). The software TMRPres2D was used to draw the prediction of transmembrane domains (Spyropoulos et al., 2004). Searches for homologous protein sequences were performed using the blastp service at the NCBI database. Multiple sequence alignments for protein sequences were done using ClustalX2 (Larkin et al., 2007).

## Results

We recently reported a mutational analysis study describing 55 random mutations of *E. chaffeensis.* The mutants were used to define the functional gene requirements for the pathogen’s growth in a physiologically relevant canine host model (Wang et al., 2020). To determine the pathogen’s requirements for its adaptation to its tick vector (*A. americanum*), we screened 45 of the prior characterized *E. chaffeensis* mutants using our recently established tick *in vivo* infection assessment model (Cheng et al., 2015; Jaworski et al., 2017) (**Table 1**). The mutants used in the current study included 25 having insertion mutations within open reading frames (ORFs) (**Table 1A**), while the remaining 20 mutations were located within intergenic regions (**Table 1B**). Thirty-two of the mutants were previously identified as essential for the *E. chaffeensis* growth and persistence in the canine host (Wang et al., 2020). Contrary to this observation, only seven mutants were recognized as critical for the bacterial tick survival as they did not persist during molting from the infected nymphs to adults (**Tables 2**, **3**), while 38 mutations had no impact on the *E. chaffeensis* presence in *A. americanum* (**Tables 4**, **5**). Four of the mutants were also previously recognized as essential for the *E. chaffeensis* growth in the canine host (**Table 2**). Three mutations having no impact on the bacterial growth in the canine host had growth defect in the tick vector (**Table 3**). Mutations in the genes encoding for *E. chaffeensis* for the biosynthesis of biotin, protein and fatty acids, DNA repair, several outer membrane proteins, and an MDR efflux pump protein did not result in the loss of the bacterial persistence in *A. americanum* (**Table 5**). Similarly, several hypothetical proteins previously identified as essential for the *E. chaffeensis* persistent growth in the canine host were found to be not critical for its growth in the tick vector (**Table 5**).

**Table 1A:** *E. chaffeensis* intragenic mutants assessed for the growth and persistence in the tick host.

Mutant	Gene*	Genomic Insertion Location	Insertion Orientation^	Insertion Location In ORF/ORFLength (bp)	Protein Identifier
**B4-1**	ECH_0665	676091	–	907/1410	Phage terminase large subunit**
**A1-2**	ECH_1067	1095813	+	695/1146	Carboxypeptidase family protein
**D5-4**	ECH_0329	317141	+	649/684	Alpha/beta hydrolase (predicted)
**D3-4**	ECH_0866	888668	+	651/993	Hypothetical protein
**S33E8**	ECH_0878	898815	–	456/1230	Hypothetical protein
**C4-2**	ECH_1038	1065224	–	5217/5892	Hypothetical protein
**C6-3^$^**	ECH_0945	964068	+	3741/4050	Polymer-forming cytoskeletal protein**
**A1-1**	ECH_0113	99608	+	924/2382	DUF3514 domain-containing protein**
**A3-2**	ECH_0187	176793	–	1154/1692	Hypothetical protein
**D4-1**	ECH_0242	226758	+	115/162	Hypothetical protein
**S33E5**	ECH_0251	236424	+	137/618	Hypothetical protein
**S34C8**	ECH_0445	423364	+	352/1191	tRNA guanosine (34) transglycosylase Tgt**
**C1-1**	ECH_0475	454669	–	1302/1347	Signal recognition particle protein
**S33C7**	ECH_0525	525880	–	1037/2001	Hypothetical protein
**B5-1**	ECH_0600	606371	+	70/144	Hypothetical protein
**D3-1a**	ECH_0614	619640	–	334/696	DUF3023 domain-containing protein**
**A4-1**	ECH_0669	683268	–	740/744	3-oxoacyl=(acyl-carrier-protein) reductase
**S34A2**	ECH_0843	861495	+	581/588	Recombination mediator RecR
**C3-2**	ECH_1127	1150113	+	117/840	Major outer membrane protein OMP-IV (P44/Msp2 family outer membrane protein**)
**B6-1**	ECH_1144	1165318	–	712/816	Major outer membrane protein P28-1 (P44/Msp2 family outer membrane protein**)
**C2-3**	ECH_0039	34759	+	1305/1647	120 kDa immunodominant surface protein (Tandem repeat effector nucleomodulin TRP120**)
**A4-3**	ECH_0561	566002	+	1781/3099	Efflux RND transporter permease subunit**
**D4-3**	ECH_0837	854867	–	56/1329	tRNA-I(6)A37 modification enzyme MiaB
**D1-4**	ECH_0104	90734	+	89/126	Hypothetical protein
**D4-4**	ECH_0666	676872	+	47/1281	Adenosylmethionine aminotransferase

**Table 1B:** *E. chaffeensis* intergenic mutants assessed for the growth and persistence in the tick host.

Mutant	Gene*	Genomic Insertion Location	Flanking Gene Orientation^	Mutation distances to flanking upstream/ downstream ORFs (bp)	Protein Identifier
**S34B1**	ECH_06570/658	671821	← + ←	120 / 26	t-RNA ser/Hypothetical protein
**D2-1**	ECH_0769/0770	779062	← - ←	414 / 59	Exopolysaccharide biosynthesis protein/ Hypothetical protein
**B5-2**	ECH_0930/0931	953314	→ + ←	243 / 158	BolA family transcriptional regulator**/Pyridoxamine 5'-phosphate oxidase
**S33A7**	ECH_1081/1082	1109518	← + →	103 / 10	SURF1 family protein/Hypothetical protein
**D1-3**	ECH_0083/0084	74911	→ - →	83 / 386	Hypothetical protein/ABC transporter permease**
**B6-3**	ECH_0579/0580	589575	→ - ←	383 / 87	Type IV secretion system protein VirB8/Hypothetical protein
**A3-3**	ECH_0699/0670	708076	→ + →	75 / 170	DUF3023 domain-containing protein**/Hypothetical protein
**A2-3**	ECH_0705/0706	716170	→ - ←	63 / 49	Peptide chain release factor 2/DUF721 domain-containing protein**
**D5-3**	ECH_0760/0761	768120	→ + ←	61 / 387	RNA polymerase sigma factor RpoD/DNA primase
**D6-3**	ECH_0995/0996	1020175	→ - →	26 / 667	Hypothetical protein/ ATP-dependent protease subunit HslV**
**B3-3**	ECH_1148/1149	1169030	→ - →	248 / 157	Hypothetical protein/preprotein translocase subunit SecA
**A2-1**	ECH_0372/0373	364550	→ + ←	356 / 159	Hypothetical Protein/Dihydroorotase
**S33E7**	ECH_0750/0751	756968	→ - →	253 / 143	DNA topoisomerase I/bifunctional ADP-dependent NAD(P)H-hydrate dehydratase/NAD(P)H-hydrate epimerase**
**A4-2**	ECH_1065/1066	1094096	← + ←	246 / 187	2-oxoglutarate dehydrogenase complex dihydrolipoyllysine-residue succinyltransferase**/gamma carbonic anhydrase family protein**
**D1-1**	ECH_0605/0606	611987	→ + →	193 / 293	Glutamyl tRNA synthetase/Hypothetical protein
**B2-3**	ECH_0149/0150	141187	← + ←	476 / 15	Pyruvate dehydrogenase complex E1 component subunit beta/Hypothetical protein
**S33F3**	ECH_0579/0580	589238	→ - ←	46 / 424	Type IV secretion system protein VirB8/ dicarboxylate/amino acid:cation symporter**
**C6-3^$^**	ECH_0894/0895	920356	← + ←	133 / 133	Kinase, pyrophosphorylase/kinase, pyrophosphorylase**
**S33B7**	ECH_0593/0594	600196	← + ←	21 / 248	Porin**/Acetylglutamate kinase
**A3-1**	ECH_0282/0283	264190	→ + ←	492 / 737	Hypothetical protein/Hypothetical protein

*E. chaffeensis gene annotations are as per the genome accession number CP000236, except when the gene annotations are updated in the revised genome annotation** and listed in the accession number NC_007799.1 (https://www.ncbi.nlm.nih.gov/); ^$^the mutant C6-3 is a mix of two independent mutants, which were cultured as a mix. ^ The + and - refer to insertion mutation in the forward orientation and in reverse orientation, respectively.

**Table 2:** *E. chaffeensis* genes essential for growth in vertebrate and tick hosts.

Mutant name	Gene Number*	Protein identifier	^#^Tick positives
A3-1	ECH_0282/0284	Hypothetical protein/Hypothetical protein	0/10
D5-4	ECH_0329^$^	Predicted as an alpha/beta hydrolase	0/10
D3-4	ECH_0866	Hypothetical protein	0/10
S33F3	ECH_0579/0581	Type IV secretion system protein VirB8-2/dicarboxylate/amino acid:cation symporter**	0/10

*E. chaffeensis gene annotations are as per the genome accession number CP000236, except when the gene annotations are updated in the revised genome annotation** and listed in the accession number NC_007799.1 (https://www.ncbi.nlm.nih.gov/); **^#^** Ticks positive/ticks tested; ^$^The blastp analysis performed at the NCBI database identified ECH_0329 protein coding sequence as having 81.86% identity with alpha/beta hydrolase of Ehrlichia canis (E-value: 8e-130).

**Table 3:** *E. chaffeensis* genes essential only for the tick host.

Mutant name	Gene Number*	Protein identifier	^#^Tick positives
S33E8	ECH_0878	Hypothetical protein (predicted to contain two internal repeats)	0/10
C6-3^$^	ECH_0945	Polymer-forming cytoskeletal protein**	0/10
C6-3^$^	ECH_0894/0895	Kinase,pyrophosphorylase/kinase, pyrophosphorylase**	0/10

*E. chaffeensis gene annotations are as per the genome accession number CP000236, except when the gene annotations are updated in the revised genome annotation** and listed in the accession number NC_007799.1 (https://www.ncbi.nlm.nih.gov/); **^#^** Ticks positive/ticks tested; ^$^The mutant C6-3 is a mix of two independent mutants which were cultured as a mix.

**Table 4:** *E. chaffeensis* mutants found as non-essential for vertebrate and tick hosts^$^.

Mutant name	Gene*	Protein identifier	^#^Tick positives
A1-1	ECH_0113	DUF3514 domain-containing protein**	8/10
B4-1	ECH_0665	Phage terminase large subunit**	4/10
A1-2	ECH_1067	Carboxypeptidase family protein	2/10
A2-1	ECH_0372/0373	Hypothetical Protein/Dihydroorotase	3/10
S33E7	ECH_0750/0751	DNA topoisomerase I/bifunctional ADP-dependent NAD(P)H-hydrate dehydratase/NAD(P)H-hydrate epimerase**	4/10
A4-2	ECH_1065/1066	2-oxoglutarate dehydrogenase complex dihydrolipoyllysine-residue succinyltransferase**/gamma carbonic anhydrase family protein**	2/10
B3-3	ECH_1148/1149	Hypothetical protein/Preprotein translocase subunit SecA	1/10
C4-2	ECH_1038	Hypothetical protein	10/10
D1-1	ECH_0605/0606	Glutamyl tRNA synthetase/Hypothetical protein	1/10
B2-3	ECH_0149/0150	Pyruvate dehydrogenase subunit Beta/Hypothetical protein	1/10

^$^Wild type E. chaffeensis was inoculated similarly in ticks to serve as the positive control; 4 of 10 ticks tested positive with wild type E. chaffeensis; *E. chaffeensis gene annotations are as per the genome accession # CP000236, except when the gene annotations are updated in the revised genome annotation** and listed in the accession # NC_007799.1 (https://www.ncbi.nlm.nih.gov/); **^#^** Ticks positive/ticks tested.

**Table 5:** *E. chaffeensis* mutants found as non-essential for the tick host which were previously found as essential for a vertebrate host (Wang et al., 2020).

Mutant name	Gene*	Protein identifier	^#^Tick positives
D1-4	ECH_0104	Hypothetical protein	1/10
A3-2	ECH_0187	Hypothetical protein	3/10
D4-1	ECH_0242	Hypothetical protein	3/10
S33E5	ECH_0251	Hypothetical protein	6/10
S33C7	ECH_0525	Hypothetical protein	9/10
B5-1	ECH_0600	Hypothetical protein	4/10
D3-1a	ECH_0614	DUF3023 domain-containing protein**	9/10
S34C8	ECH_0445	tRNA guanosine(34) transglycosylase Tgt**	8/10
C1-1	ECH_0475	Signal recognition particle protein	1/10
A4-1	ECH_0669	3-oxoacyl=(acyl-carrier-protein) reductase	1/10
S34A2	ECH_0843	Recombination mediator RecR	1/10
C3-2	ECH_1127	Major outer membrane protein OMP-IV (P44/Msp2 family outer membrane protein**)	3/10
B6-1	ECH_1144	Major outer membrane protein P28-1 (P44/Msp2 family outer membrane protein**)	1/10
C2-3	ECH_0039	120 kDa immunodominant surface protein (Tandem repeat effector nucleomodulin TRP120**)	4/10
A4-3	ECH_0561	Efflux RND transporter permease subunit**	1/10
D4-3	ECH_0837	tRNA-i(6)A37 modification enzyme MiaB	6/10
D4-4	ECH_0666	Adenosylmethionine aminotransferase	1/10
S33B7	ECH_0593/0594	Porin**/Acetylglutamate kinase	10/10
S34B1	ECH_657/658	t-RNA ser/Hypothetical protein	2/10
D2-1	ECH_0769/0770	Exopolysaccharide biosynthesis protein/Hypothetical protein	7/10
B5-2	ECH_0930/0931	BolA family transcriptional regulator**/Pyridoxamine 5’-phosohate oxidase	1/10
S33A7	ECH_1081/1082	SURF1 family protein/Hypothetical protein	10/10
D1-3	ECH_0083/0084	Hypothetical protein/ABC transporter permease**	
B6-3	ECH_0579/0580	Type IV secretion system protein VirB8/Hypothetical protein	10/10
A3-3	ECH_0699/0670	DUF3023 domain-containing protein**/Hypothetical protein	1/10
A2-3	ECH_0705/0706	Peptide chain release factor 2/DUF721 domain-containing protein**	1/10
D5-3	ECH_0760/0761	RNA polymerase sigma factor RpoD/DNA primase	6/10
D6-3	ECH_0995/0996	Hypothetical protein/ATP-dependent protease subunit HslV**	4/10

*E. chaffeensis gene annotations are as per the genome accession number CP000236, except when the gene annotations are **updated in the revised genome accession number NC_007799.1 (https://www.ncbi.nlm.nih.gov/); **^#^** Ticks positive/ticks tested.

**Figure 1:**
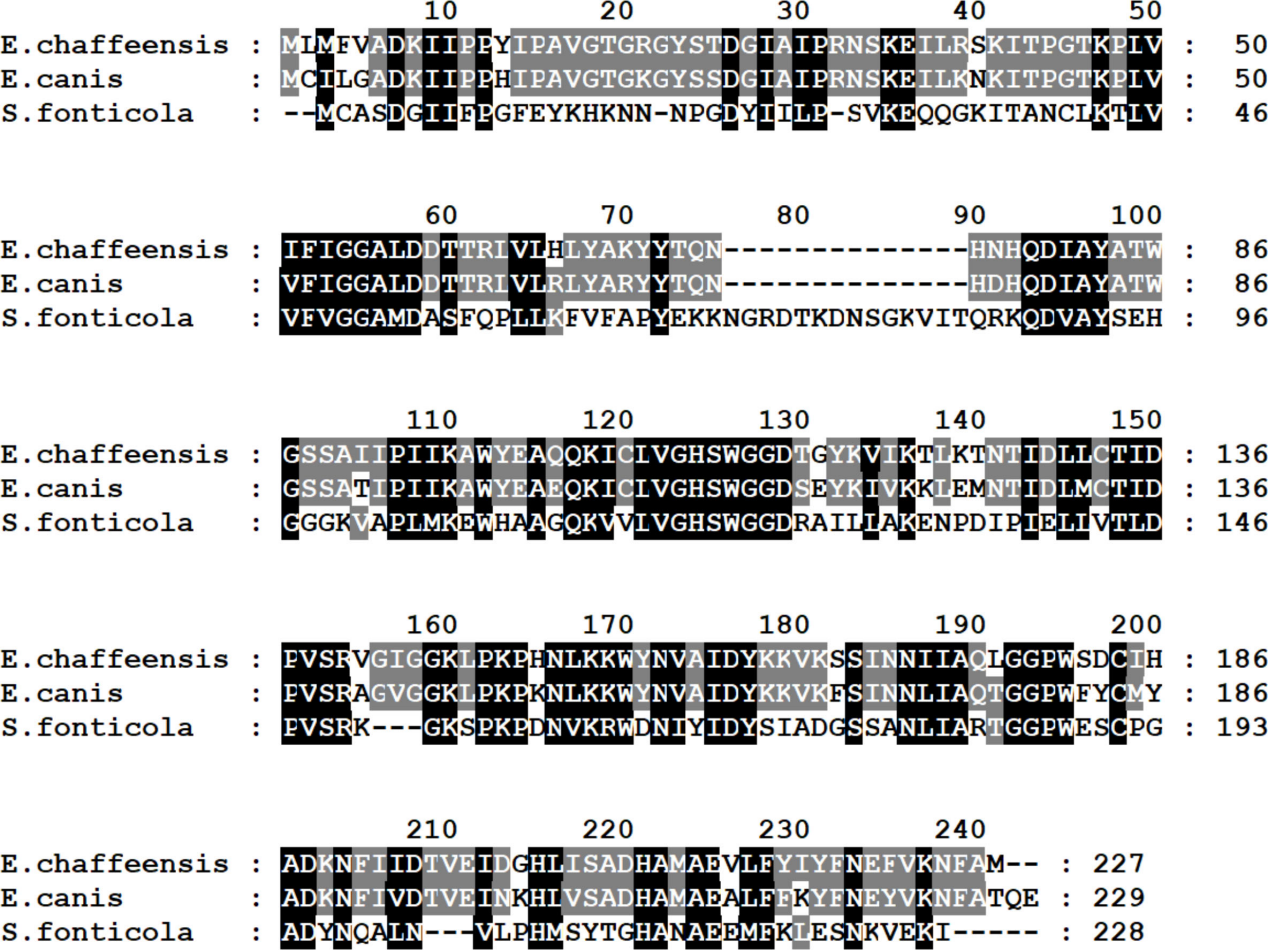
Alignment of the predicted alpha/beta hydrolase of *E. chaffeensis* (GenBank # WP_006011217) encoded by gene ECH_0329, *E. canis* (GenBank # WP_011304785) and *Serratia fonticola* (GenBank # WP_202728975). Identical amino acids are shaded. Amino acid numbers of each protein are shown on the right.

**Figure 2:**
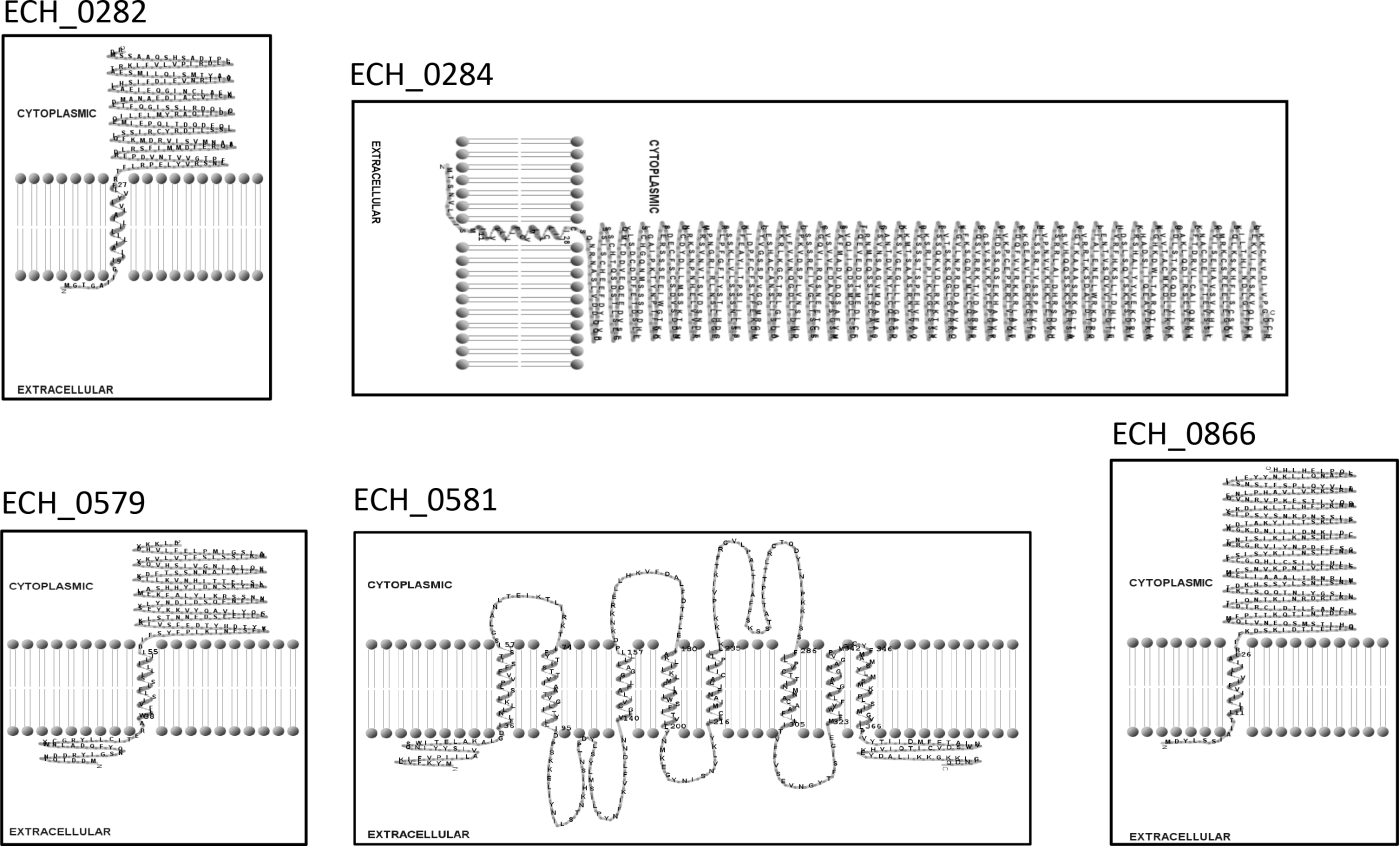
Transmembrane segments for five proteins of *E. chaffeensis* encoded by ECH_0282, ECH_0284, ECH_0579, ECH_0581 and ECH_0866 which were predicted by PRED-TMR (Pasquier et al., 1999). The software TMR Pres2D (Spyropoulos et al., 2004) was used to draw the predicted transmembrane domain.

Mutation within the ORF of ECH_0329 coding for a predicted alpha/beta hydrolase (**Figure 1**) caused growth defect of the organism in both the tick vector and in the canine host (**Table 2**). The remaining three mutations causing rapid clearance from the tick and canine hosts (**Table 2**) were within or near the genes identified as coding for hypothetical proteins (ECH_0866, ECH_0282 and 0284), a T4SS protein VirB8-2 (ECH_0579), and dicarboxylate amino acid:cation symporter protein (ECH_0581). Analysis using the PRED-TMR (a transmembrane protein prediction tool) (Pasquier et al., 1999; Spyropoulos et al., 2004), predicted the ORFs of ECH_0282, ECH_0284 and ECH_0866 as having one transmembrane domain each located at N-terminal regions (**Figure 2**). The second intergenic mutation is located downstream to the coding region of VirB8-2 (ECH_0579) and upstream to dicarboxylate amino acid:cation symporter protein (ECH_0581). PRED-TMR similarly predicted VirB8-2 as having one transmembrane domain at N-terminal. Dicarboxylate amino acid:cation symporter proteins is predicted to contain 8 transmembrane domains distributed throughout the protein sequence (Pasquier et al., 1999; Spyropoulos et al., 2004) (**Figure 2**).

**Figure 3:**
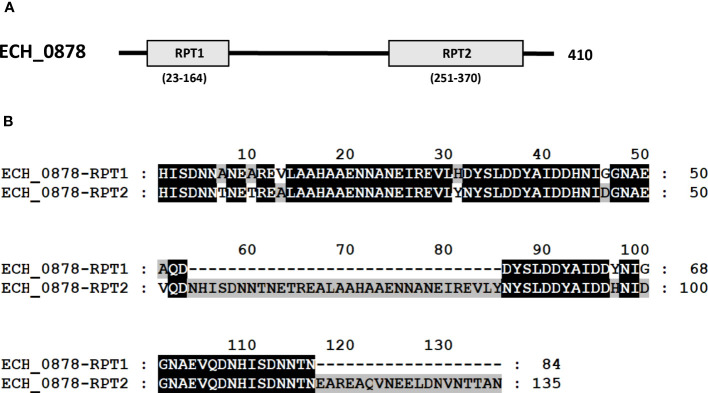
*E chaffeensis* ECH_0878 ORF was analyzed using the SMART protein and conserved domain search analysis (Letunic et al., 2020). **(A)** A scheme described that the protein encoded by gene ECH_0878 is identified to contain two major repeats, RPT1 and RPT2. **(B)** Alignment of two repeats of the protein encoded by gene ECH_0878.

**Figure 4:**
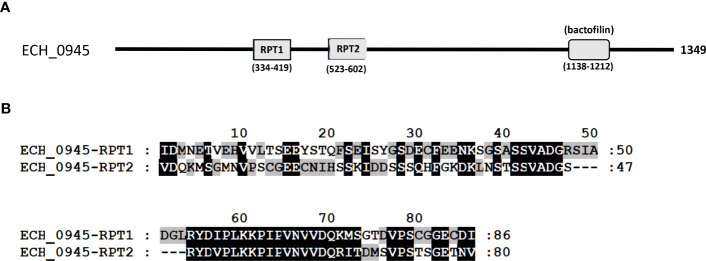
The polymer-forming cytoskeletal protein of *E chaffeensis* encoded by ECH_0945 was analyzed by SMART protein and conserved domain search (Letunic et al., 2020). **(A)** A scheme described that the protein encoded by gene ECH_0945 is identified to contain two major repeats (RPT1 and RPT2) and a conserved bactofilin. **(B)** Alignment of two repeats of the polymer-forming cytoskeletal protein encoded by gene ECH_0945.

Three mutants causing failed growth of *E. chaffeensis* (**Table 3**) only in the tick vector represented one each insertion mutation within hypothetical protein genes; ECH_0878 and ECH_0945 and the 3^rd^ mutation located between genes ECH_0894 and ECH_0895 encoding for partial ORFs for a kinase/pyrophosphorylase (GenBank # NC_007799.1). The SMART protein and conserved domain search analysis (Letunic et al., 2020) found ECH_0878 ORF as having two highly homologous repeats with 64% sequence identity (**Figure 3**). Similarly, ECH_0945 codes for a polymer-forming cytoskeletal protein having two internal repeat sequences with 38% identity. This gene also has a similarity to bactofilin (**Figure 4**).

## Discussion

During the last four decades, several tick-transmitted rickettsial pathogens have been recognized as causing diseases in people and several vertebrate hosts (Rodino et al., 2020). *E. chaffeensis* is first identified in 1987 as a human pathogen transmitted from *A. americanum* tick and followed by the discovery of additional rickettsial pathogen infections caused by *A. phagocytophilum, E. ewingii* and *E. muris eauclairensis* (Dawson et al., 1991; Chen et al., 1994; Buller et al., 1999; Pritt et al., 2017). Despite several recent advances, much remains to be understood regarding how these broad host range rickettsial pathogens have evolved in adapting to vertebrate and tick hosts. Progress in molecular genetic studies focused on disrupting genes and their use to define the importance of rickettsial pathogens is very limited (Cheng et al., 2013; Crosby et al., 2015; McClure et al., 2017; Wang et al., 2020). Such studies are important in discovering proteins critical for host-pathogen interactions and in devising effective methods of controlling infections (Liu et al., 2007; Chen et al., 2012; Cheng et al., 2013; Crosby et al., 2014; Crosby et al., 2015; Nair et al., 2015; Lamason et al., 2018; Bekebrede et al., 2020; Wang et al., 2020; Hove et al., 2022). Despite low mutation efficiency in *Ehrlichia* and *Anaplasma* species, we recently reported the development of a small library of mutants for *E. chaffeensis* and described its value in identifying many genes critical for the bacterial persistent growth in a physiologically relevant vertebrate host infection model (Wang et al., 2020). Our prior studies demonstrated that function disruption mutations in many pathogen genes can be detrimental to *E. chaffeensis* persistence in the canine host, while not having an impact on the *in vitro* growth conditions (Cheng et al., 2013; McGill et al., 2016; Wang et al., 2020). The prior studies also demonstrated that the pathogen is exposed to unique challenges during its growth *in vivo* and that it requires many functional genes to facilitate its adaptation and persistence in a vertebrate host.

Considering our prior published evidence documenting that *E. chaffeensis* protein expression is highly variable during its replication in vertebrate and tick host background (Singu et al., 2005; Singu et al., 2006; Seo et al., 2008), in the current study we tested the hypothesis that the pathogen has unique functional gene requirements. Specifically, we investigated which *E. chaffeensis* genes were critical for the pathogen’s growth in its tick host; *A. americanum*. We observed remarkable differences in the *E. chaffeensis* genes required for its growth in the tick compared to the vertebrate host. We utilized our previously well-established needle infection method to screen 45 *E. chaffeensis* mutants for their abilities to infect *A. americanum* nymphs and their transstadial transmission during molting to the adult stage (Cheng et al., 2015; Jaworski et al., 2017). We observed that only 7 mutations in *E. chaffeensis* caused growth impact for the pathogen in *A. americanum*, unlike the documentation of many mutations as essential for the bacterial persistence in the canine host (Wang et al., 2020). Notably, 28 mutations caused growth defects of *E. chaffeensis* in the canine host grew normally in *A. americanum* suggesting that *E. chaffeensis* adaptation strategies differ for the tick compared to the vertebrate host. *E. chaffeensis* genes essential for its continued growth in a vertebrate host included genes encoding for three immunodominant membrane proteins; the 120 kDa protein and two p28 Omps (OMP 1V and p28-1) and those involved in the biosynthesis of biotin, protein and fatty acids, DNA repair and MDR efflux pump. Mutations in all these genes, however, did not prevent the bacterial replication within its tick host. While different membrane protein expressions are anticipated, it is not clear how the deficiency resulting from mutations in several metabolic genes of *E. chaffeensis* is compensated during its replication and transstadial transmission in the tick. Possible explanations are that the deficiency of the metabolic pathway proteins is compensated from other sources within the tick. Hard ticks cohost numerous pathogenic and non-pathogenic bacteria, including several rickettsial endosymbionts (Wikel, 2018; Rodino et al., 2020; Hirunkanokpun et al., 2022; Qi et al., 2022). Recently, Wang et al. described the detailed overview of how ticks and other blood sucking ectoparasites depend on symbiotic microorganisms for completing essential aspects of their biology, such as development and reproduction (Wang et al., 2023). Endosymbionts and other microorganisms co-existing in ticks may likely also benefit from each other. The deficiencies resulting from mutations leading to the deprivation of essential metabolites and proteins in *E. chaffeensis* are possibly compensated by other bacteria which coexist in *A. americanum*. This hypothesis, however, remains to be tested.

The bacterial survival with deficiency for the immunodominant outer membrane proteins may reflect that the three previously identified membrane proteins are only critical for the *E. chaffeensis* growth in the vertebrate host. These data are consistent with the previous report demonstrating that the two p28-Omp proteins (OMP 1V and p28-1) expressed in a vertebrate host are not among the *E. chaffeensis* proteins found in the tick host (Unver et al., 2002). Of the three mutants that were exclusively absent in the tick vector, one of the mutations is located near a gene (ECH_0895) which encodes for a kinase/pyrophosphorylase. This protein is among the outer membrane expressed proteins of *E. chaffeensis* when it is cultured in tick cells (ISE6) (Seo et al., 2008). Another mutation within ECH_0945 gene codes for polymer-forming cytoskeletal protein having high homology to bactofilins. Bactofilins are known to have diverse functions, including cell stalk formation to chromosome segregation and motility in bacteria (Deng et al., 2019). Together, the unique differences noted in the *E. chaffeensis* outer membrane-expressed proteins suggest that the pathogen has distinct needs for the cell surface expressed proteins when replicating in the tick host compared to a vertebrate host.

A mutation in the ECH_0329 gene coding for an alpha/beta hydrolase caused rapid clearance from both vertebrate and tick hosts. This protein is expressed on the *E. chaffeensis* morula membrane during the bacterial replication in a macrophage cell line (Kondethimmanahalli and Ganta, 2022) and another alpha/beta hydrolase (ECH_0326 gene product) is present on the bacterial outer membrane (Seo et al., 2008). Alpha/beta hydrolases are a large family of proteins with many known functions and are present in several pathogenic bacteria. For example, *Mycobacterium tuberculosis* has 105 alpha/beta hydrolases, and they are involved in lipid metabolism, evasion and modulation of immune responses, detoxification, and metabolic adaptations including growth (Yang et al., 2014; Johnson, 2017). These enzymes in *M. tuberculosis* are known to contribute to the bacterial virulence (Johnson, 2017). The clearance of *E. chaffeensis* alpha/beta hydrolase mutant suggests that this multifunctional enzyme and likely its homologs may be important for the bacterial survival in both vertebrate and tick hosts.

Additional mutations causing the pathogen clearance from *A. americanum* included those present in intergenic locations. They included mutations near genes coding for proteins having transmembrane domains, such as the protein encoded from ECH_0282 gene, T4SS protein VirB8-2 (ECH_0579), dicarboxylate amino acid:cation symporter (ECH_0581), and kinase/pyrophosphorylase (ECH_0895). The dicarboxylate amino acid:cation symporter is critical for the uptake of C(4)-dicarboxylates, such as succinate or l-malate, in bacteria (Youn et al., 2009). Similarly, T4SS-associated proteins are critical for supporting the bacterial evasion of host responses mediated by the release of T4SS effectors into infected host cells (Rikihisa, 2021; Lin et al., 2023). While it is unclear how the intergenic mutations described may have impacted the functions of genes 5’ and 3’ to insertion sites, our previously published detailed investigation demonstrates that such mutations, as well as some intragenic mutations, cause polar effects impacting gene expressions from genes upstream and/or down to the mutated regions (Cheng et al., 2015). For example, an intergenic mutation downstream to ECH_0230 prevented the transcription from the gene and caused *in vivo* growth defect. A mutation between ECH_0479 and ECH_0480 caused the transcriptionally silent ECH_0480 gene to be transcriptionally active; intergenic mutations between ECH_0202 and ECH_0203 and between ECH_0284 and ECH_0285 caused RNA expression enhancement from ECH_0203 and ECH_0285, respectively. Similarly, mutations within ECH_0379 and ECH_0660 resulted in preventing transcription from these genes as well as the genes located 5’ to them; ECH_0378 and ECH_0659, respectively. These two mutations also caused the bacterial growth defect in vertebrate hosts. Thus, it is not surprising that the intergenic mutations have detrimental outcomes for the bacterium’s *in vivo* growth. Intergenic space sequences typically include promoter regions and other regulating domains where small non-coding RNAs and DNA binding proteins bind and contribute to transcriptional regulation (Wassarman, 2002; Raghavan et al., 2011; Schroeder et al., 2015). The functional roles of all the predicted essential membrane expressed proteins remains to be determined for *E. chaffeensis* replication in both vertebrate and tick hosts.

Considering the exposure of outer membrane domains to host cells, the detection of several bacterial membrane-bound proteins of *E. chaffeensis* as essential for the pathogen’s continued survival in vertebrate and tick hosts will be valuable for furthering our understanding of bacterial pathogenesis, host-pathogen interactions and in devising effective methods of control and prevention. In particular, very little information is currently available regarding what bacterial factors define the survival of *E. chaffeensis* and other related tick-borne rickettsiales in dual host environments and in causing pathogenesis. This mutational analysis study is the first to identify many *E. chaffeensis* genes uniquely required for its adaptation to tick and vertebrate hosts and that the data will be valuable for advancing our understanding of the bacterial host-pathogen interactions.

## Data availability statement

The datasets presented in this article are not readily available. Requests to access the datasets should be directed to romanganta@missouri.edu.

## Author contributions

DJ, YW, AN, HL, and RG contributed to experimental design and execution, drafting, and finalizing the manuscript. RG conceived the project and secured funding. All authors contributed to the article and approved the submitted version.
